# Aortic Arch Replacement without Deep Hypothermic Circulatory Arrest

**DOI:** 10.1155/2021/8821182

**Published:** 2021-01-06

**Authors:** Zviad Bakhutashvili, Lia Janelidze, Kakhaber Beria, Simon Matikashvili, Eduard Limonjiani

**Affiliations:** Chapidze Emergency Cardiology Center, Georgia

## Abstract

A 60-year-old man presented with a thoracic aortic aneurysm without rupture accompanied by severe nonrheumatic aortic valve insufficiency and unstable angina. Surgery was performed and included several steps: (1) resection and reconstruction of ascending aorta and aortic arch using a tube graft, (2) replacement of aortic valve using a biological prosthesis, and (3) coronary artery bypass grafting was performed with two distal anastomoses. All of these procedures were performed with total cardiopulmonary bypass without deep hypothermic circulatory arrest under conditions of moderate hypothermia using dual concurrent cannulation of the subclavian and femoral arteries.

## 1. Introduction

The critical consideration in approaching aortic arch surgery is the way in which to best protect the brain while providing surgical access to the cerebral vessels. This issue remains the subject of controversy and research and involves two key aspects: (1) minimizing cerebral ischemia and (2) preventing cerebral embolization of air and atheromatous debris. The methods for preventing cerebral ischemia, such as hypothermic circulatory arrest (HCA), selective antegrade perfusion (SACP), and retrograde cerebral perfusion (RCP), are widely used [1]. Each of them has advantages and disadvantages. In our case, a long operation was performed without circulatory arrest at moderate (32°C) hypothermia with bilateral antegrade cerebral perfusion. Unilateral cerebral arterial perfusion only took five minutes while the anastomosis was performed between graft and the left carotid artery. Cerebral oximetry was monitored, and no abnormal changes were noted.

## 2. Case Description

**Figure 1:**
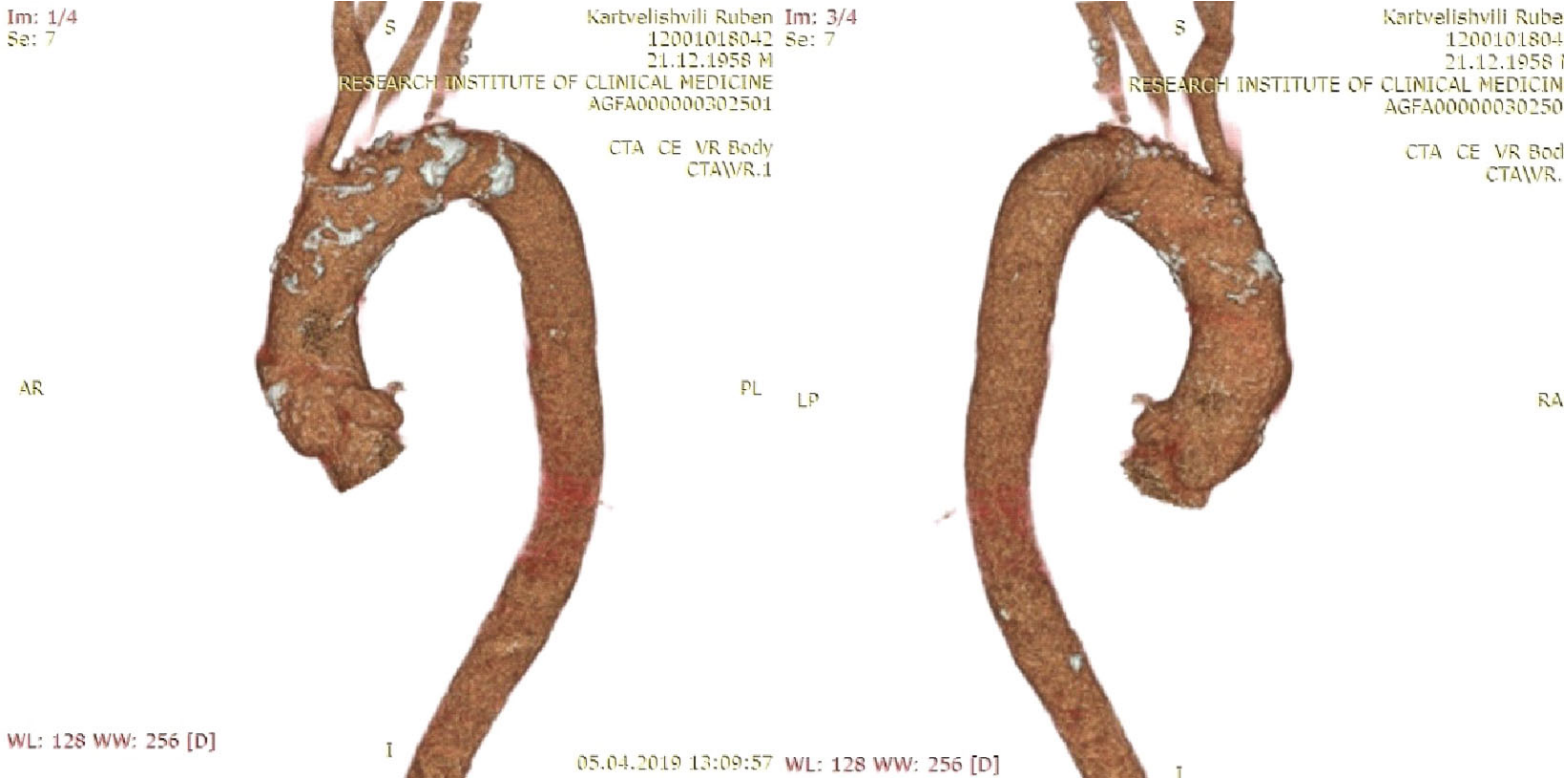
Computed tomography angiography.

Our patient presented with symptoms that included fatigue, shortness of breathing during exercise, and dyspnea. Physical examination revealed severe aortic insufficiency (color Doppler jet 65%; effective regurgitant orifice area [EROA] = 0.3 cm^2^), left ventricular end − diastolic diameter (LVEDD) = 6 cm, left ventricular end − systolic diameter (LVESD) = 4.3 cm, ejection fraction (EF) = 58%. Selective coronarography revealed diffusive polytopic lesions. Computed tomography (CT) results are shown in Figure 1 and showed root = 3.8, ascending aorta = 4 cm, and arch = 3.8 cm in addition to typical syphilitic aortic aneurysm with extensive calcified plaques involving the ascending aortic arch and brachiocephalic arteries [2]. Surgery was scheduled.

### 2.1. Surgical Steps

**Figure 2:**
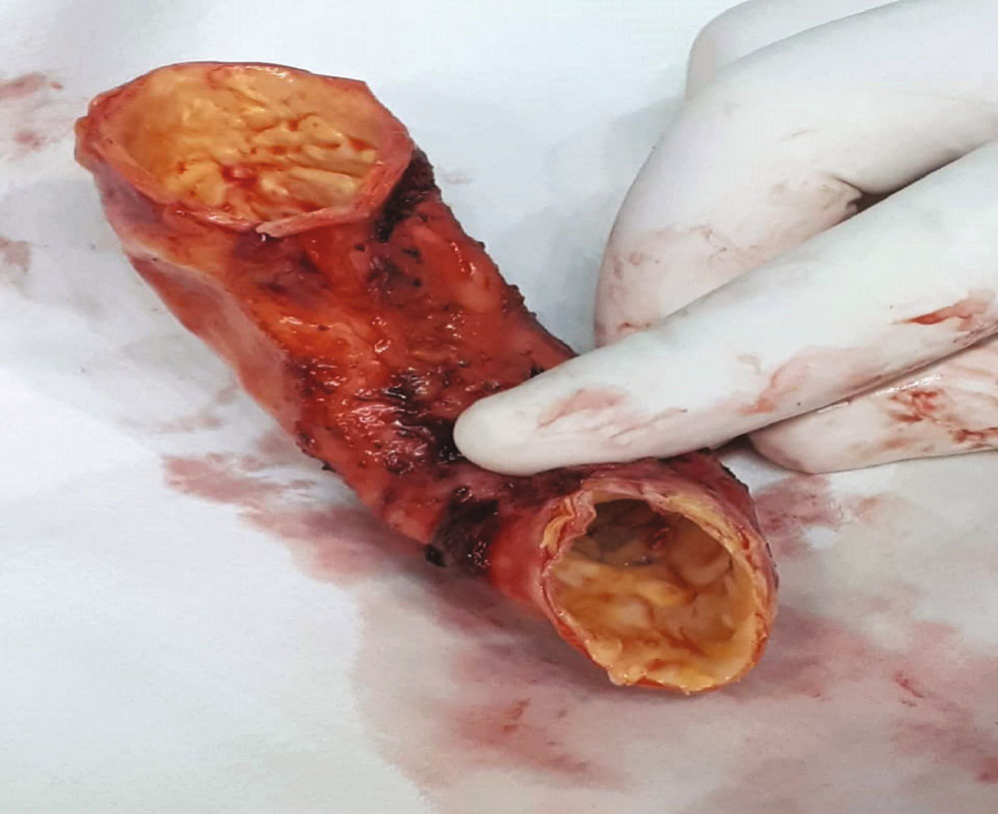
The intima develops wrinkle ridges and plaques described as a “tree bark appearance.”

A median sternotomy and pericardiotomy were performed. By inspection, the ascending aorta and arch with branches had extensive calcified plaques Figure 2. Right subclavian and femoral arteries were cannulated using the Seldinger method. A two-stage venous cannula was placed through the right atrium. Cardiopulmonary bypass was initiated under conditions of moderate hypothermia (32°C). A vent for the left heart was placed through the right superior pulmonary vein and then through the mitral valve into the left ventricle. Once the aorta has been crossclamped, the ascending aorta was opened, and antegrade tepid blood cardioplegia was administered directly into the coronary orifices. Cardioplegia was repeated at 20 to 30 min intervals during the operation.

For coronary revascularization, two distal (sequential) anastomoses were performed using a greater saphenous vein. In the first stage, end-to-side anastomosis between the vein and obtuse marginal (OM) branch was done followed by a perpendicular sequential side-to-side procedure between the left anterior descending artery (LAD) and saphenous vein.

The aortic valve cusps were incised with scissors, and the calcified deposits were removed with a scalped and rongeur. Once debridement was complete, the annulus was sized with a valve sizer, and a MEDTRONIC-N25 bioprosthetic aortic valve was secured to the annulus using 15 double-needled interrupted pledged sutures.

The ascending aorta, arch, and brachiocephalic arteries were exposed. The distal limit for resecting the descending aorta free from calcified deposits was identified and mobilized circumferentially for future clumping. The bifurcated prosthetic graft [3] was used for restoring brachiocephalic arteries completeness beginning with the left subclavian artery and innominate artery followed by the left carotid artery. Anterior cerebral perfusion (ACP) was administered through both carotid arteries. After five minutes, unilateral selective cerebral perfusion (SCP) via the right subclavian was added while anastomosis between the graft and left carotid artery was being done. During the operation, especially during the process mentioned above, near-infrared spectroscopy (NIRS) was monitored carefully, and no abnormal changes were noticed.

**Figure 3:**
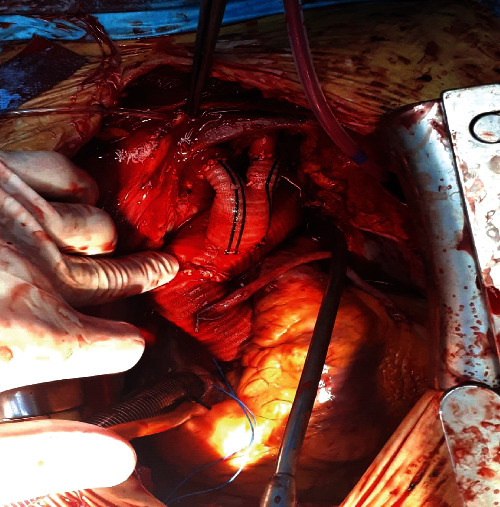
Aortic arch after repair.

Once brachiocephalic artery integrity was accomplished, the ascending aorta, arch, and unhealthy tissue of the descending aorta were resected and reconstructed using a 28 mm tube graft. The bifurcated prosthetic graft used for brachiocephalic arteries wholeness was implanted into the ascending artery tube graft Figure 3.

Aorta was declamped, de-airing was performed, and proximal anastomosis was performed between the greater saphenous vein and ascending aortic graft. Cumulative bypass time was 222 min, and cumulative crossclamp time was 151 min. The patient recovered well without temporary neurological dysfunction (TND) and was discharged on the seventh postoperative day.

## 3. Discussion

Two basic mechanisms can lead to ischemia cerebral injury during thoracic aorta surgery: (1) stroke is the first type of injury and has received the most attention, mainly because of its devastating consequences. These ischemic infarcts are detectable by conventional imaging techniques and result from embolic events that are thought to be independent of the method of brain protection, and (2) the second type of injury results from focal or global ischemia resulting from interrupted or inadequate blood flow, thus giving rise to the clinical syndrome, TND, which is characterized by varying degrees of obtundation, confusion, agitation, or transient Parkinsonism. It is now generally accepted that TND is a direct consequence of inadequate cerebral protection and therefore related to the method of protection used. Our approach yielded outstanding results. After the operation was finished, the patient awakened in less than 12 h and started following our instructions. As mentioned above, no signs of TND were noted. This type of surgery needs multispecialistic involvement at the time of surgery and during the admission to the intensive care unit. With the emerging endovascular technologies, hybrid procedures became more and more widely used. Needless to mention that postoperative thromboembolism prevention is of paramount importance [4]. Deep vein thrombosis is the cornerstone of venous thromboembolism [4]; therefore, deep vein thrombosis should be prevented by different means of prevention, such as low-dose subcutaneous unfractionated heparin (UFH) or low molecular-weight heparin (LMWH). Both are estimated to reduce venous thromboembolism incidence by 50%. Multispecialty involvement is the key when dealing with such complicated cases.

## Data Availability

Data are available upon request.
